# BiliCheck vs JM-103 in identifying neonates not at risk of hyperbilirubinaemia

**DOI:** 10.1186/1824-7288-39-46

**Published:** 2013-07-23

**Authors:** Costantino Romagnoli, Piero Catenazzi, Giovanni Barone, Lucia Giordano, Riccardo Riccardi, Antonio Alberto Zuppa, Enrico Zecca

**Affiliations:** 1 Division of Neonatology, Department of Pediatrics, Catholic University Sacred Heart, Rome, Italy; 2 Division of Neonatology, Policlinico A. Gemelli, Catholic University Sacred Heart, Largo A. Gemelli, 8, 00168, Rome, Italy

**Keywords:** Newborn, Neonatal jaundice, Transcutaneous bilirubin measurements

## Abstract

**Background:**

Transcutaneous bilirubinometry is widely used to predict hyperbilirubinemia by using several devices. The aim of this study was to compare the predictive ability of BiliCheck vs JM-103 in identifying neonates not at risk of significant hyperbilirubinemia, putting the data obtained with the two instruments on our transcutaneous bilirubin nomogram built with the BiliCheck.

**Methods:**

Transcutaneous bilirubin (TcB) measurement was performed when jaundice appeared in newborn babies and/or just before discharge from the hospital. It was performed at the forehead with the two instruments within 5 minutes by two experienced neonatologists, each one blind to the value obtained by the other. Blood samples were drawn to obtain total serum bilirubin (TSB) levels soon after TcB measurements.

**Results:**

A total of 627 paired-sample measurements were obtained from 298 newborn babies. Out of the total population studied, 16 newborn babies (5.4%) showed significant hyperbilirubinemia defined as TSB value >17 mg/dL, or as need for phototherapy treatment according to the AAP guidelines. TcB measurements showed false negative results in the first 60 hours of life using both devices. After the 60^th^ hour of life, TcB measurements using both devices successfully predicted newborn babies not at risk of significant hyperbilirubinemia, being the JM-103 more reliable than BC because of fewer false positive results.

**Conclusions:**

Our study shows that both BC and JM-103 can exclude subsequent significant hyperbilirubinemia when the measurements are performed after the 60^th^ hour of life. Nevertheless, the transcutaneous pre-discharge screening should be considered only as the first step, and it has to be followed by a follow-up through the first days after discharge.

## Background

Early discharge of healthy late preterm and full term neonates from hospital influenced an increase in hospital readmission rates due to hyperbilirubinemia, and even the more serious reappearance of kernicterus [1,2]. Visual assessment of jaundice and risk factors evaluation did not give positive results in predicting subsequent hyperbilirubinemia [3,4], while plotting total serum bilirubin (TSB) measurements on hour-specific nomograms allowed the reduction in hospital readmission rate for severe hyperbilirubinemia (TSB > 30 mg/dl) [5-8]. The suitability of transcutaneous determination of bilirubin (TcB) to reduce both the need and adverse effects of TSB measurement has been widely documented [9-15]. Several hour-specific nomograms for TcB have been developed [14,16-20] but few of them have been prospectively validated [21,22]. In the past years we developed a skin bilirubin nomogram for the first 96 h of life in a European normal healthy population using BiliCheck™([BC] Respironics, Marietta, GA – USA) and then validated it in a prospective, observational multicenter study [18,22]. BC and JM-103 (Drager Medical Inc, Telford, Pennsylvania) reliability has been evaluated in two recently published papers [23,24]. The purpose of this study was to compare the predictive ability of BiliCheck vs JM-103 in identifying neonates not at risk of significant hyperbilirubinemia, putting the data obtained with the two instruments on our transcutaneous bilirubin nomogram built with the BiliCheck.

## Methods

### Study population

This prospective cohort study was conducted through January to July 2011 in a single maternity ward.Ethics committee of the Department of Paeditrics approved the study and the consent form. Informed consent was obtained from both parents. The inclusion criteria were gestational age ≥35 weeks (based on ultrasound assessment in the first trimester, if available or on postmenstrual age otherwise), absence of congenital malformation and of any disease requiring an admission to the neonatal intensive care unit. Intramuscular administration of 1 mg vitamin K was the only drug administered to studied infants. Breast or bottle feeding every 3 hours was started within one hour of life.

### Measurement of TcB and TSB

The measurement of TcB was performed on each baby either when he/she was visually jaundiced and/or just before discharge from the hospital. The determination of TcB was made at the forehead with the two instruments within 5 minutes, according to manufacturer instructions. BC was calibrated before each measurement using a BiliCal™, and each measurement was performed with 5 readings in different points of neonatal forehead. JM- 103 was used on the forehead performing 5 readings too. Reading locations for both devices were distanced from the hairline and free of any bruising, nevus, haemangioma or other skin anomalies. Each device was used by one neonatologist unaware of the other one’s results and of the TSB level. A blood sample (50 μl) for TSB measurement was obtained by heel stick puncture, soon after TcB measurement. Capillary tubes were protected from ambient light and were assayed with direct spectrophotometer (Microbilimeter Dual Beam Plus model, Ginevri, Rome, Italy) after centrifugation. A total of 627 paired-sample measurements were obtained from 298 newborn babies.

### Follow-up of studied infants

Newborn babies were never discharged before 72 h of age, independently from the mode of delivery. All newborn babies with a pre-discharge TSB value > 75^th^ percentile of our nomogram were discharged only after two consecutive decreasing TSB values, 12 hours apart, making us able to identify the peak TSB level. Newborn infants with pre-discharge TSB level between the 50^th^ and the 75^th^ percentile were discharged and controlled 48 h later for hyperbilirubinemia using TSB measurement. Parents of infants with TSB <50^th^ percentile were counseled to return to the hospital within 5 days, or earlier if they observed a persistent jaundice. The decision to use phototherapy was made by the attending neonatologist according to AAP guidelines [25]. For babies exposed to phototherapy we considered only pre-treatment measurements. All perinatal data were recorded in a single database with a selected log of any event occurring during the study period.

### Outcome

Measurements of TcB were plotted on our percentile-based hour-specific nomogram separately by two researchers after completion of the study. Significant hyperbilirubinemia was defined as TSB value >17 mg/dL, or as need for phototherapy treatment according to the AAP guidelines. Newborn babies with TcB value below the 75^th^ percentile that developed subsequent significant hyperbilirubinemia were defined as false negative results.

### Statistical analysis

In our previous experience, BC showed to be 96% sensitive using the 75^th^ percentile of our nomogram [22]. Sample size and power analysis were calculated assuming that JM-103 would be at least 90% sensitive to be considered equivalent. Calculations derived a sample size of 564 paired measurements to yield 80% power with alfa 0.05.

We used the Student’s t-test for continuous predictors and the Fisher’s test for categorical data. We calculated the sensitivity, specificity, positive predictive and negative values plotting TcB data against the 75^th^ percentile of our TcB nomogram because this percentile resulted the best predictor in a previous multicenter study performed on our population. Receiver operating characteristic (ROC) curve analysis was performed with SPSS software, which was used to assess the predictive ability of the two studied devices. ROC curves were compared using the Hanley & McNeil method.

## Results

A total of 298 newborn babies (143 males and 155 females, 279 full term and 19 late preterm) were enrolled in the study (Table 1). Mean gestational age (±SD) was 38.7 (1.4) weeks (range: 35–42) and mean birth weight (BW) was 3200 (440) g (range: 1830–4430). The majority of babies were Caucasian (92.3%) and were born after spontaneous delivery (58.4%). Breast and/or bottle feeding were the prevalent feeding methods, only 34.9% received exclusive breastfeeding. Delayed meconium passage (>24 h of life) was observed in 20 (6.7%) babies, and only 12 (4.0%) had a weight loss greater than 10%. Out of the total studied population, 16 newborn babies (5.4%) were diagnosed as having significant hyperbilirubinemia, and 9 (3.0%) received phototherapy. None of the babies required an exchange transfusion. No cases of significant hyperbilirubinemia were noticed among not jaundiced babies. The mean variation coefficient for BC and JM-103 was 7.4% ± 1% and 6.9% ± 1.2%, respectively.

**Table 1:** Baseline characteristics of the study population

**Variables**	**Neonates (298)**
**Gestational age (wks)**	**38**.**7 ± 1**.**4**
>**37**	**279 (93.6)**
** 35 - 36**	**19 (6.4)**
**Birth weight (g)**	**3200 ± 440**
**Male**	**143 (48.0)**
**Mode of delivery**	
** Vaginal**	**174 (58.4)**
** Cesarean section**	**117 (39.3)**
** Ventouse**	**7 (2.3)**
**Feeding**	
** Exclusive breast feeding**	**104 (34.9)**
** Breast + bottle feeding**	**160 (53.7)**
** Bottle feeding**	**34 (11.4)**
**Delayed meconium passage (>24 hrs)**	**20 (6.7)**
**Weight loss >10 %**	**12 (4.0)**
**Significant hyperbilirubinemia**	**16 (5.4)**
**Required phototherapy**	**9 (3.0)**
**TSB >17 mg/dL**	**7 (2.3)**

Values expressed as mean ± SD or number (%).

Table 2 shows the predictive ability of the 75^th^ percentile of our TcB nomogram to identify babies with significant hyperbilirubinemia using the TcB measurements obtained by BC and JM-103. TcB measurements using both the devices showed false negative results in the first 60 hours of life. In this period, BC values were more reliable than JM-103 values because of less false negatives, but with more false positives. After the 60^th^ hour of life there were no false negative results for both the devices, but JM-103 was more reliable because of less false positive results.

**Table 2:** **Ability of JM-103 and BC measurements below the 75th percentile of TcB nomogram (ref. **[22]**) to predict significant hyperbilirubinemia, for designated time periods**

**Hours of age**	**TP (n)**	**FN (n)**	**TN (n)**	**FP (n)**	**Sensitivity (%)**	**Specificity (%)**	**PPV (%)**	**NPV (%)**
**JM**-**103**								
**24 to 48 hours**	2	2	124	47	50.0	72.5	4.1	98.4
**49 to 60 hours**	4	1	79	21	80.0	79.0	16.0	98.8
**61 to 72 hours**	5	0	129	37	100	77.7	11.9	100
**73 to 96 hours**	13	0	117	46	100	71.8	22.0	100
**Total**	24	3	449	151	88.9	74.8	13.7	99.3
**Bilicheck**								
**24 to 48 hours**	3	1	73	98	75.0	42.7	3.0	98.6
**49 to 60 hours**	4	1	45	55	80.0	45.0	6.8	97.8
**61 to 72 hours**	5	0	82	84	100	49.4	5.6	100
**73 to 96 hours**	13	0	73	90	100	44.8	12.6	100
**Total**	25	2	273	327	92.6	45.5	7.1	99.3

*TP* True Positive, *FN* False Negative, *TN* True negative, *FP* False Positive, *PPV* Positive Predictive Value, *NPV* Negative Predictive Value.

**Figure 1:**
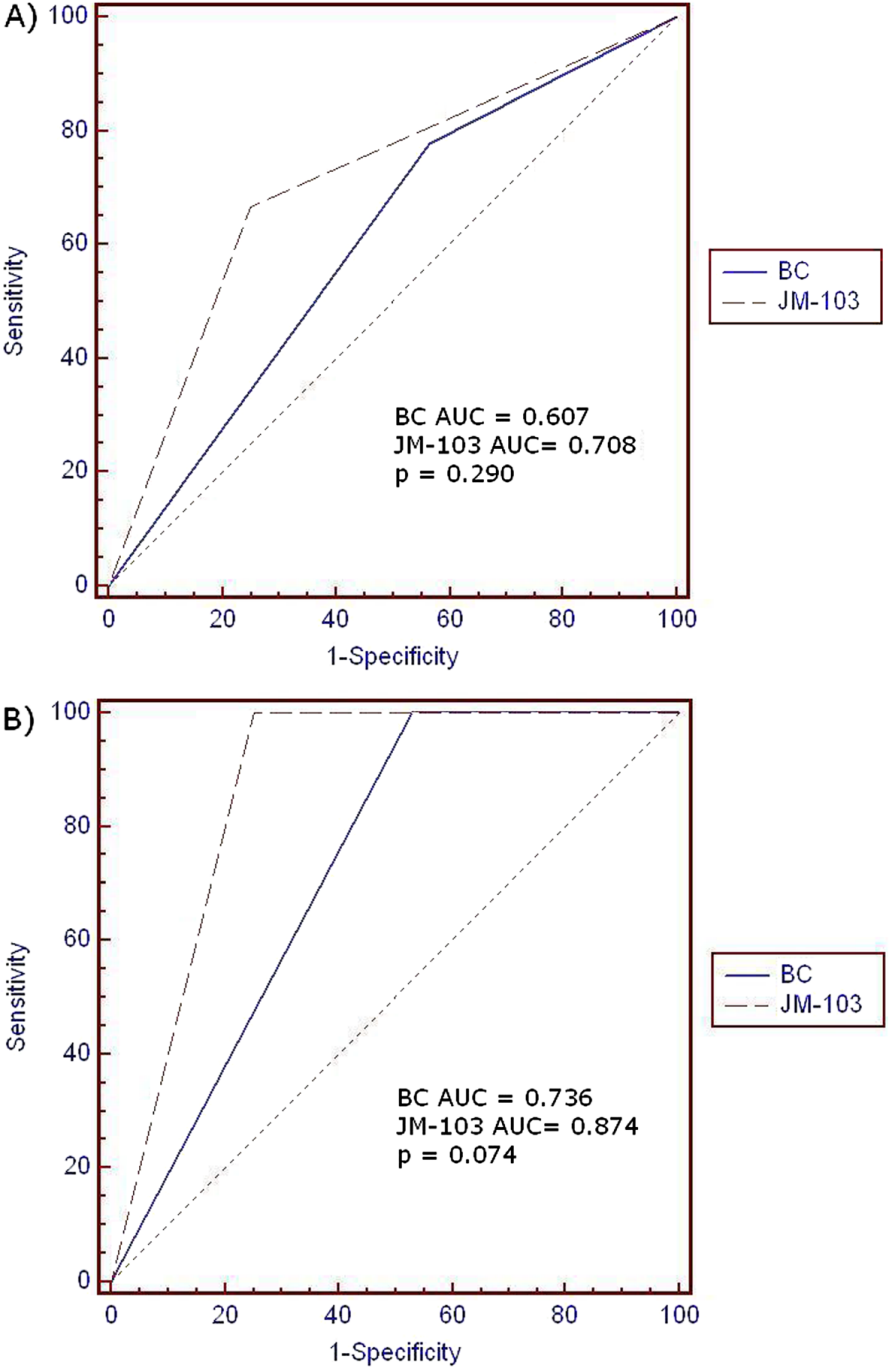
**ROC curves during different hours of age: A) 24–60 h; B) 61–96.** Full line and dashed line represent the BC and the JM-103 performance, respectively.

The ROC curve for the 75^th^ percentile of TcB is shown in Figure 1 where the true positive rate (sensitivity) was plotted in function of the false positive rate (1-specificity). The area under the ROC curve (AUC) measured the accuracy of the percentiles in predicting significant hyperbilirubinemia using JM-103 or BC. AUCs for both the devices were lower in the first 24–60 hours of life than between 61–96 hours. JM-103 had higher AUC than BC between 61–96 hours, and this difference borders statistical significance (p = 0.074).

In order to calculate the execution times of both procedures for a subset of 50 newborn babies, a second investigator clocked times for TcB measurements. Mean time for one TcB measurement was 41.2 (5.0) sec (range: 38–55 sec) for BC and 19.2 (2.6) sec (range: 16–25 sec) for JM-103. The longer time for BC was mainly due to the calibration procedure.

## Discussion

Even if healthy term newborns are discharged in many countries before the 60^th^ hour of life, this burdening problem is not yet clearly defined [26]. The Expert Committee for Severe Neonatal Hyperbilirubinemia and European Society for Pediatric Research and American Academy of Pediatrics published detailed recommendations emphasizing the importance of universal, systematic assessment of the risk of severe hyperbilirubinemia, suggesting that a systems-based approach to prevent severe neonatal hyperbilirubinemia should be implemented at all birthing facilities and coordinated with continuing ambulatory care [27]. Therefore, the identification of newborn babies at risk of severe hyperbilirubinemia is still one of the most challenging problems for the neonatologist. The use of TSB measurement before discharge from the hospital reduced the number of babies with very high levels of bilirubin [5-8], but apparently it did not reduce the incidence of bilirubin-induced neurological damage [28]. TcB use to avoid TSB measurement, reducing the pain and risks of this procedure, progressively increased during the last years [9-15]. We recently validated our TcB nomogram in a large Italian prospective study, showing that 100% of sensitivity can be obtained with TcB measurements only after 48 hours of life, using the 75^th^ percentile [22]. However, two recent papers from South Carolina and Canada raised some concerns because of false-negative results from pre-discharge TcB screening [29,30]. Moreover, it is not known if the hour specific TcB nomogram developed with measurements obtained with the BC could have the same predictivity when a different transcutaneous bilirubinometer is used. The present study was performed for this reason, in order to compare the predictive ability of BC vs JM-103 using our transcutaneous bilirubin nomogram built with the BC.

Our experience showed that 100% sensitivity was obtained after 60 hours of life for both devices, giving JM-103 less false positive results. In the first 60 hours of life there were false negative results with both devices (2 with BC and 3 with JM-103) but JM-103 had less false positive results. Furthermore, JM-103 was less time consuming and less expensive because of the absence of consuming materials.

Our data ask for caution when TcB measurements and TcB nomogram are used as predictive tools if an early (<60 hours) discharge from the hospital is performed. While our previous experience with BC showed the best predictive power after 48 hours of life, in this present work we had one false negative with both the devices between 48 and 60 hours of life. These false negative results would have not been identified even if the risk factors for hyperbilirubinemia suggested by Keren et al. and by Maisels et al. were applied [3,21]. In fact, both the infants were white, males, spontaneously delivered at 36 and 37 weeks of gestation, and had a normal clinical course in the first 72 hours of life. They were breast and bottle fed and had a normal weight loss (<7%). They were discharged at 72 hours of life and were controlled 48 hours later reaching their maximum bilirubin level (17.2 mg/dL) at 85 and 98 hours of life.

This study has some limitations: it is not a population-based study because the enrolment was based on clinical practice, it includes a vast majority of Caucasian newborn babies, and the incidence of significant hyperbilirubinemia is low. Their strengths are its robust design and the high follow-up rate for enrolled newborn babies. All babies referred to our ambulatory care facility for clinical evaluation after discharge, and no case of readmission for hyperbilirubinemia was noticed.

## Conclusions

Our study shows that BC and JM-103 can be used for the TcB measurements to exclude significant hyperbilirubinemia after the 60^th^ hour of life. Nevertheless, our experience suggests that the pre-discharge screening for hyperbilirubinemia should be considered only as the first step of a long route that ends only after an accurate follow-up through the first days after discharge. Therefore, we agree with Fay et al. [26] when they state that the follow-up for hyperbilirubinemia should not be discouraged by risk screening practice, without exposing newborn babies to unnecessary and unjustified risks.

## Competing interests

The authors declare that they have no competing interests.

## Authors’ contributions

CR and EZ designed the study and have made substantial contributions in drafting manuscript. PC, LG, and RR performed data acquisition and validation. GB and PC contributed to interpretation of data and performed statistical analysis. AAZ verified data analysis and revised the manuscript critically. All authors read and approved the final manuscript.
